# OVERCOMING RECRUITMENT APPROACHES TO IDENTIFY PARTICIPANTS WITH DEMENTIA

**DOI:** 10.1093/geroni/igac059.1533

**Published:** 2022-12-20

**Authors:** Elizabeth Galik

**Affiliations:** University of Maryland, Baltimore, Baltimore, Maryland, United States

## Abstract

Clinically the work up for dementia often includes a history and physical, neuropsychiatric screening measures and neuroimaging. These assessments are neither practical nor realistic when identifying participants for research studies. To confirm a diagnosis of dementia for study participants in the FFC-AC-EIT study we developed a measurement model. The model included four measures: the AD8, the Functional Activities Questionnaire, the Clinical Dementia Rating Scale, and the Saint Louis University Mental Status Examination. In the first 346 patients consented, 176 were enrolled and 158 were ineligible. The mean age of the participants was 80.70 (SD=9.60) and the majority were female (64%) and white (66%). There was evidence of reliability based on internal consistency and construct validity based on model fit using Rasch analysis and Structural Equation Modeling. All four measures are recommended as a pragmatic way in which to comprehensively determine evidence of dementia for research studies.

